# Case Report: A new case of *YARS1*-associated autosomal recessive disorder with compound heterozygous and concurrent 47, XXY

**DOI:** 10.3389/fped.2023.1282253

**Published:** 2023-12-06

**Authors:** Janene Kuan, Ashleigh Hansen, Hua Wang

**Affiliations:** ^1^ Department of Pediatrics, University of California, San Francisco, CA, United States; ^2^ Division of Genetics, Department of Pediatrics, Loma Linda University School of Medicine, Loma Linda, CA, United States; ^3^ Neurosciences Department, Cedars-Sinai, Los Angeles, CA, United States

**Keywords:** tyrosyl-tRNA synthetase 1 (YARS1), autosomal recessive disorder, whole exome sequencing, multisystem disease, 47, XXY, Charcot-Marie-Tooth disease, IMNEPD2

## Abstract

Aminoacyl-tRNA synthetases play a pivotal role in catalyzing the precise coupling of amino acids with their corresponding tRNAs. Among them, Tyrosyl tRNA synthetase, encoded by the YARS1 gene, facilitates the aminoacylation of tyrosine to its designated tRNA. Heterozygous variants in the YARS1 gene have been linked to autosomal dominant Charcot-Marie-Tooth type C, while recent findings have unveiled biallelic YARS1 variants leading to an autosomal recessive multisystemic disorder in several cases. In this report, we present a novel case characterized by dysmorphic facies, and multisystemic symptoms, prominently encompassing neurological issues and a microarray conducted shortly after birth revealed 47, XXY. Utilizing whole exome sequencing, we uncovered a paternally inherited likely pathogenic variant (c.1099C > T, p.Arg367Trp), previously reported, coinciding with the father's history of hearing loss and neurological symptoms. Additionally, a maternally inherited variant of uncertain significance (c.782T > G, p.Leu261Arg), previously unreported, was identified within the YARS1 gene. The observed phenotypes and the presence of compound heterozygous results align with the diagnosis of an autosomal recessive disorder associated with YARS1. Through our cases, the boundaries of this emerging clinical entity are broadened. This instance underscores the significance of comprehensive genetic testing in patients exhibiting intricate phenotypes.

## Introduction

Aminoacyl-tRNA synthetases (ARS) constitute a group of enzymes that catalyze the vital process of coupling amino acids with their respective tRNAs. This linkage, determined by the tRNA's anticodon, represents a crucial precursor to mRNA translation into polypeptides within both the cytoplasm and mitochondria. Within the human genome are 17 cytoplasmic ARSs, 17 mitochondrial ARS enzymes, and three bifunctional enzymes facilitating tRNA charging in both cellular compartments (1). While all ARS genes share a common catalytic motif for tRNA aminoacylation, select genes have acquired supplementary noncatalytic domains and roles in various cellular processes throughout evolution. For instance, YARS1 (tyrosyl-tRNA synthetase 1) splits into fragments through proteolysis, acting as a cytokine with angiogenic and leukocyte chemoattractant properties (2). GARS1 (glycyl-tRNA synthetase 1) contributes to anti-tumorigenic defenses (3), HARS1 (histidyl-tRNA synthetase 1) participates in the inflammatory response within inflammatory myositis (4), LARS1 (leucyl-tRNA synthetase 1) orchestrates protein synthesis and autophagy for cellular growth (5), and WARS1 (tryptophanyl-tRNA synthetase 1) is engaged in angiogenesis (6). Additionally, several ARS assemble into complexes that manage cellular organization, translation regulation, and non-translational ARS functions (7). The critical role of ARS is underscored by the identification of 56 human genetic diseases linked to ARS gene variants as of 2022, encompassing 46 autosomal recessive biallelic disorders and 10 autosomal dominant monoallelic disorders (2).

Specifically, cytoplasmic tyrosyl-tRNA synthetase, encoded by the YARS1 gene, orchestrates the aminoacylation of tyrosine onto its corresponding tRNA. Heterozygous YARS1 variants have been associated with autosomal dominant Charcot-Marie-Tooth type C (8). Recently, several cases have unveiled the involvement of biallelic pathogenic YARS1 variants in an autosomal recessive multisystemic disorder characterized by failure to thrive, developmental delay, muscular hypotonia, liver dysfunction, pulmonary disease, and hearing impairment. Different YARS1 variants result in slightly distinct phenotypes. In this study, we present a novel case of this autosomal recessive YARS1-associated disorder, characterized by compound heterozygous variants.

## Case description

**Figure 1:**
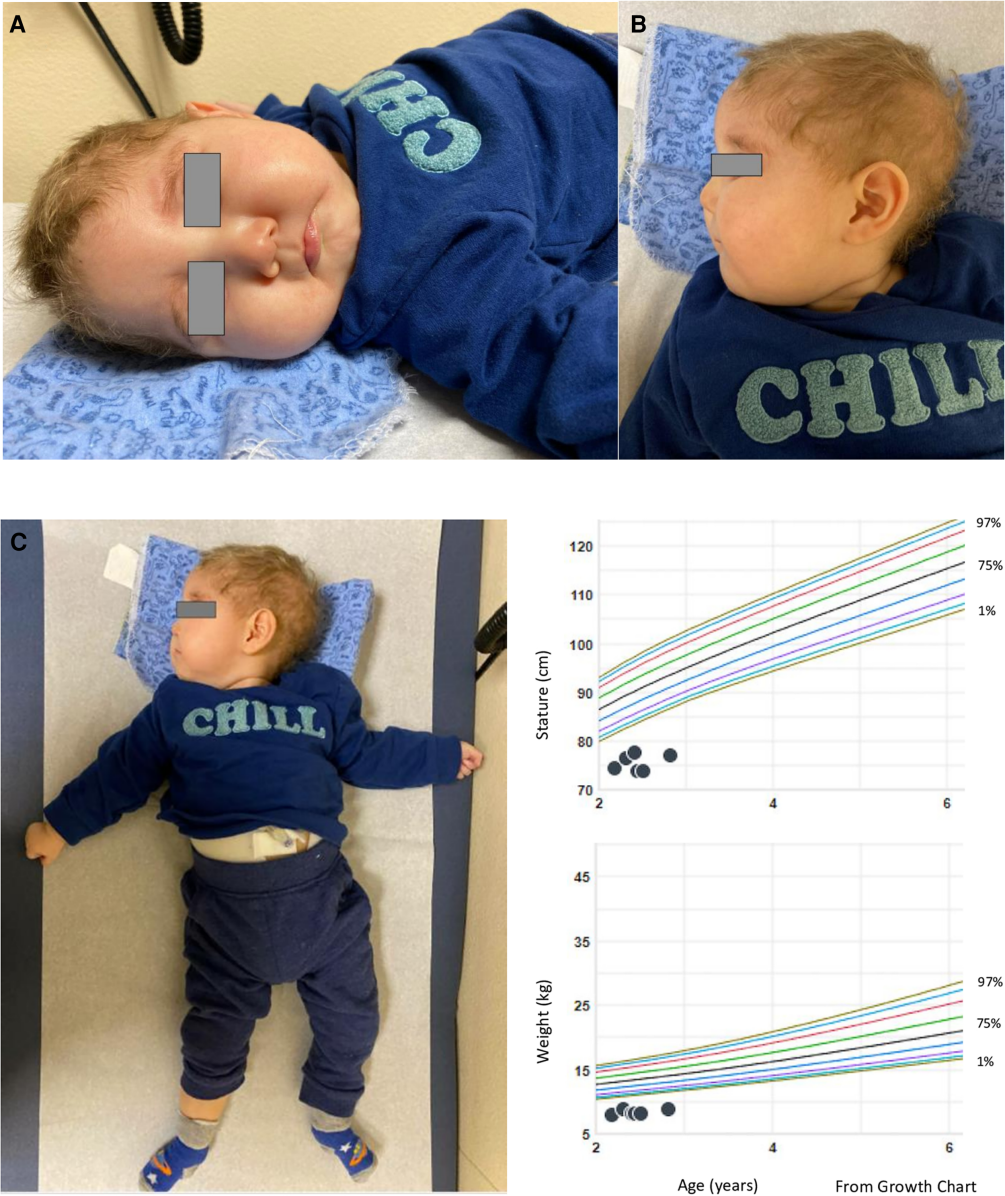
(**A–C**) photographs of the patient (at age of 16 months). In (**A**) the patient's photographs reveal pronounced microcephaly and distinctive dysmorphic facial features, characterized by a narrow forehead, prominent cheeks, a broad nasal bridge, and deeply set eyes. Notably, nystagmus was also observed (not shown). (**B**) Depicts the patient's normal ears. In (**C**) it is evident that both length and weight measurements fall below the 1%ile in growth chart.

**Figure 2:**
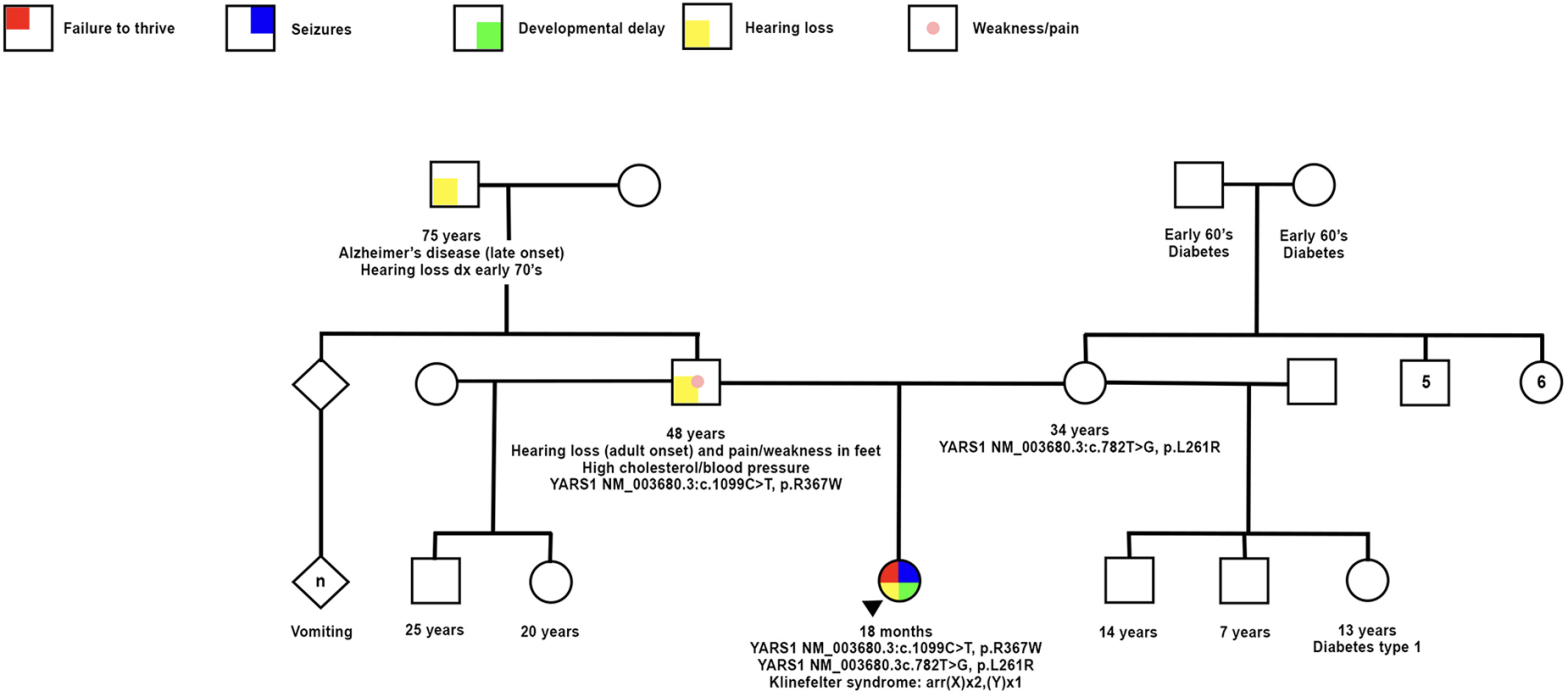
Family history documentation showing the proband and symptoms with positive genetic testing for compound heterozygous variants *YARS1* p.Arg367Trp and p.Leu261Arg, as well as klinefelter syndrome confirmed by chromosome microarray. Mother and father with respective genetic testing and associated phenotypes.

This case involves a Hispanic male who was delivered at 37 weeks through normal spontaneous vaginal delivery without any complications. The initial admission occurred at 12 weeks of age, prompted by a history of poor feeding persisting for 6 weeks, accompanied by non-bloody, nonbilious emesis for the same duration. Additionally, the patient experienced 2 weeks of intermittent seizure-like activity characterized by jerking movements and a blank stare. The vomiting episodes were forceful and occurred after nearly every feeding, ejecting both through his mouth and nose, involving the entire feeding. The seizure-like activity manifested as repetitive movements involving both arms and the face, lasting up to 30 s, occurring 1–3 times daily, and unrelated to the vomiting episodes. Prior to this admission, the patient had multiple emergency room visits due to emesis and seizure-like activity. EEG results indicated focal sites of hyperexcitability, while brain MRI yielded no significant findings. During this admission, intermittent hyperkalemia was detected. The ACTH stimulation test yielded normal results, and a review of the newborn screen was negative. An abdominal ultrasound revealed an echogenic liver suggestive of possible hepatocellular dysfunction. Initial AST and ALT levels were within the normal range, but subsequent values showed elevation. An echocardiogram revealed a patent foramen ovale with a moderate-sized shunt. The brain MRI exhibited subtle CSF intensity related to the right cardiothalamic groove and the wall of the right lateral ventricle, accompanied by mild right lateral ventricular enlargement, possibly indicative of prior germinal matrix hemorrhage. A chromosome microarray was recommended during the initial genetics consultation, revealing 47, XXY (Klinefelter syndrome). The patient was discharged after the resolution of seizures with Keppra and improvement in feeding through a nasogastric (NG) tube. Notably, he did not pass his initial or subsequent newborn hearing screening in the left ear. Subsequent consultations with his primary care physician identified microcephaly, hypotonia, and significant developmental delays, leading to referrals for physical therapy (PT) and occupational therapy (OT). Due to repeated instances of removing the NG tube and failure to thrive, a percutaneous endoscopic gastrostomy (PEG) tube was placed. At 10 months of age, the patient was referred to our pediatric genetics clinic due to his severe developmental delay and generalized hypotonia. Physical examination at the initial and follow up visit (at 16 months) revealed microcephaly and distinctive dysmorphic facial features, including a narrow forehead, prominent cheeks, a wide nasal bridge, and deep-set eyes (see Figures 1A,B). His length and weight measurements were both below the 1st percentile (see Figure 1C). Nystagmus was also observed. Upon thorough investigation, the family history unfolded with details about the patient's 48-year-old father, encompassing a medical background featuring high cholesterol, high blood pressure, eczema, and psoriasis. Notably, the father grappled with adult-onset hearing loss, relying on hearing aids. Additionally, he reported sensations of pain and weakness in his feet. Further contributing to the familial health narrative, the paternal grandfather was noted to contend with both hearing loss and Alzheimer's disease. Additional details in the family history reveal instances of vomiting in a paternal cousin. Despite both parents being Hispanic, the family explicitly denied any consanguinity (see Figure 2. Pedigree).

### Whole exome sequencing

**Figure 3:**
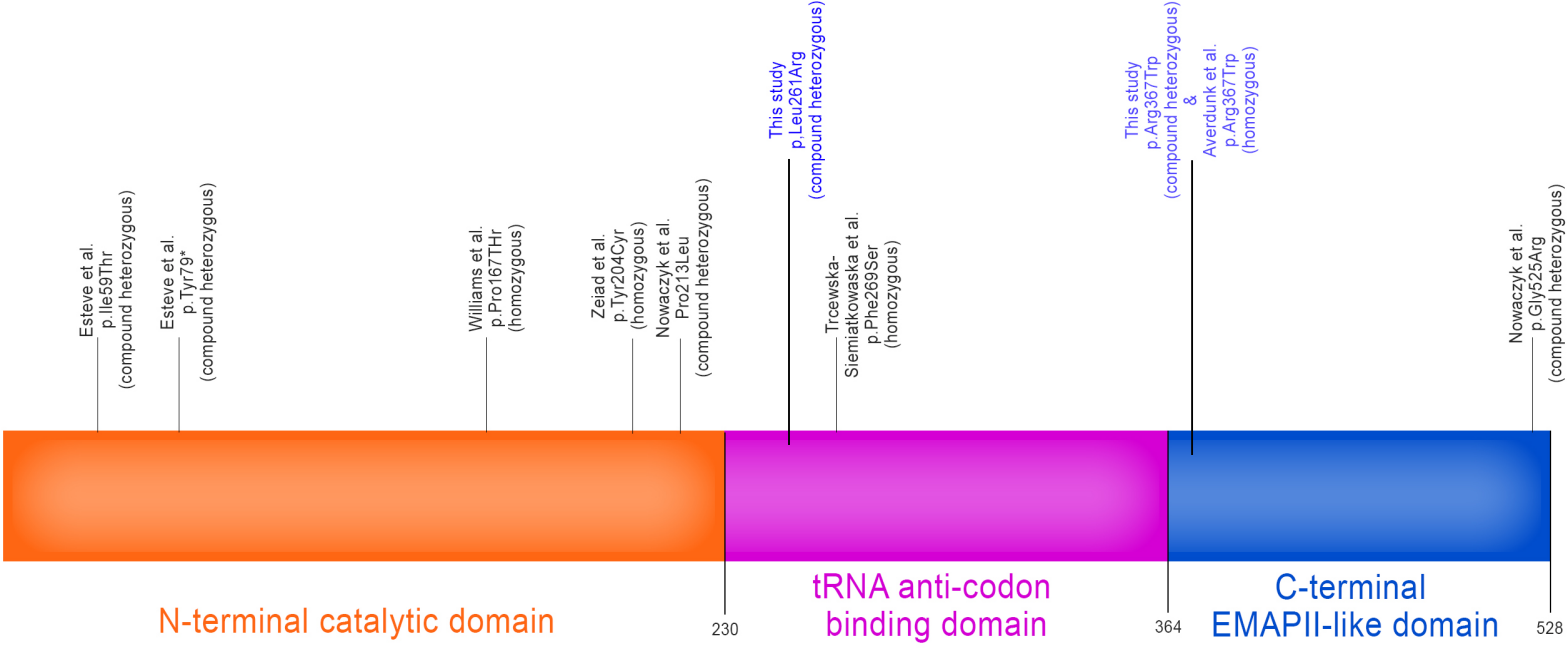
*YARS1* gene map showing 3 protein domains: catalytic N-terminal domain, tRNA anti-codon binding domain and the C-terminal EMAP-II-like domain. Associated *YARS1* homozygous and compound heterozygous variants implicated in autosomal recessive multisystemic disease involving failure to thrive, developmental delay, muscular hypotonia, liver dysfunction, pulmonary disease, and hearing impairment as has been reported in the literature to date. Variants listed in this study are in blue. [Authorized adaptation from (9)].

Whole exome sequencing was conducted at Baylor Genetics using the Illumina Dragen BioIT Platform (accession number in the report: G918060950). Variants were interpreted following ACMG guidelines and patient phenotypes. The results unveiled a likely pathogenic variant (c.1099C > T, p.Arg367Trp, paternally inherited) and a variant of uncertain significance (c.782T > G, Leu.L261Arg, maternally inherited) in the YARS1 gene (NM_003680.3) (See Figure 3). The YARS1 p.Arg367Trp variant has been described in ClinVar (ID:567612) which was first reported by Adverdunk (9) as homozygous variant. It is exceedingly rare in the healthy population (<0.001% in gnomAD). It is a missense variant predicted to be deleterious (CADD: 32.000), located within the tRNA anticodon binding domain, and is classified as pathogenic due to its high evolutionary conservation. The maternally inherited YARS1 p.Leu261Arg variant has not been previously reported in ClinVar and is categorized as uncertain significance. It is predicted to be deleterious (CADD: 32.000) and resides in a region displaying high evolutionary conservation within the C-terminal EMAPII-like domain. This variant has low frequency in healthy populations (gnomAD <0.001%). Multiple prediction tools, including SIFT (which assigns a “deleterious supporting” evidence) and Polyphen (which assigns “deleterious moderate”), predict its deleterious nature, while FATHMM assigns an uncertain score. Although the amino acid change is one that retains hydrophobicity (leucine to arginine), there is considerable size difference between the two residues, with leucine being smaller thus providing evidence for functional conformation change of tertiary and quaternary protein structures (from PubChem). Based on the clinical phenotypes and the findings from whole exome sequencing, a diagnosis of YARS1-associated autosomal recessive disorder was established. Subsequent investigations revealed systemic involvement. Laboratory tests indicated anemia, an elevated white blood cell count of 25.83 bil/l (normal range: 4.8–11.8 bil/l), with lymphocytes accounting for 75% (normal range: 30%–70%). Additionally, abnormal liver function and unusual plasma amino acid levels were observed.

## Discussion

YARS1, situated on chromosome 1 and comprised of 13 exons, is not only involved in aminoacylation but has also been implicated in gastric cancer as a potential tumorigenic factor (10) and plays a role in angiogenesis (6). Heterozygous mutations in YARS1 have been linked to dominant intermediate Charcot-Marie-Tooth (DI-CMT) disease (OMIM#118220), initially identified by Jordanoval et al. (11). These pathogenic variants in YARS1 exhibit a gain-of-function effect, leading to heightened interactions with nuclear TRIM28. This interaction triggers a transcriptional response that subsequently alters neurodevelopment, dendrite morphogenesis, and glucose metabolism (12). It is noteworthy that the manifestations of ARS-associated CMT are primarily attributed to length-dependent axonal degeneration rather than demyelination (13). In the case of YARS1-associated CMT, upper extremity weakness and atrophy, along with hyperreflexia, have been reported (12). This presentation differs from other forms of CMT, which typically feature lower extremity-predominant muscle weakness and atrophy.

Bi-allelic variants in YARS1 have led to a newly defined clinical entity, first documented by Nowaczyk et al. in 2017. In their study, Nowaczyk et al. reported on two siblings who were compound heterozygous for likely pathogenic variants, specifically c.638C > T, p.(Pro213Leu), and c.1573G > A, p.(Gly525Arg) in YARS1. Both siblings exhibited failure to thrive and hypotonia, but only one of them presented with mild developmental delay, liver dysfunction, cystic lung disease, and corpus callosum thinning (14). Subsequently, Tracewska-Siemiątkowska et al. in 2017 described an individual with a homozygous variant c.806 T > C, p.(Phe269Ser) who additionally experienced severe visual impairment due to progressive rod-cone generation, primary amenorrhea, thin corpus callosum, and thrombocytosis. Remarkably, unlike other cases involving developmental delay, this patient exhibited normal psychomotor development (15). In 2019, Williams et al. reported on seven related children from an Amish community who were homozygous for the YARS1 variant c.499C > A, p.(Pro167Thr). These children presented with a broader spectrum of symptoms, including brain dysmyelination, nystagmus, exocrine pancreatic insufficiency, renal dysfunction, hypoglycemia, anemia, intermittent proteinuria, and recurrent bloodstream infections (16). Averdunk et al. (9) identified 12 individuals from six families with a recurrent homozygous missense variant c.1099C > T, p.(Arg367Trp) in YARS1, who also exhibited microcephaly, short stature, ataxia, microcytic anemia, and hypothyroidism. A subset of individuals additionally suffered from hearing impairment, gastroesophageal reflux, vomiting, and pulmonary disease (9). In 2021, Zeiad et al. described an infant with a novel homozygous variant of uncertain significance, c.611A > C, p.(Tyr204Cys) in YARS1, who additionally presented with hyperinsulinemic hypoglycemia, exocrine pancreatic insufficiency, primary hypothyroidism, recurrent infections, anemia, and coagulopathy (17). Finally, Estève et al. (8) reported on a patient heterozygous for two novel variants in YARS1, c.176T > C, p.(Ile59Thr), and c.237C > G, p.(Tyr79*), with the former variant located in the highly conserved aminoacylation domain of YARS1. This female patient exhibited a more severe phenotype, including failure to thrive, developmental delay, global hypotonia, liver disease, pulmonary fibrosis, chronic diarrhea, anemia, and microcephaly (8).

In OMIM (OMIM #619418), YARS1-associated autosomal recessive disorder is designated as infantile-onset multisystem neurologic, endocrine, and pancreatic disease-2 (IMNEPD2). This condition is characterized by a set of common features, including failure to thrive, developmental delay, muscular hypotonia, liver dysfunction, pulmonary disease, and hearing impairment. Most reported cases, including our own, exhibit these common features, with the notable exception of lung disease in our case. Among the dysmorphic features frequently observed in these cases are deep-set eyes. Additionally, other reported characteristics encompass prominent cheeks, an elongated and narrow forehead, a wide nasal bridge, a hanging columella, a flat philtrum, microretrognathia, an open mouth appearance, and facial weakness (refer to Table 1). Our case notably exhibits a majority of these characteristic facial features.

**Table 1:** Phenotypes of individuals with *YARS1* variants [Authorized adaptation from (8)].

Case report		This study	Estève et al.	Zeiad et al.	Averdunk et al. (12 patients)	Williams et al. (7 patients)	Tracewska-Siemiątkowska et al.	Nowaczyk et al. (2 siblings)	Frequency
*YARS1* variants		p.Leu261Arg p.Arg367Trp	p.Ile59Thr and p.Tyr79*	p.Tyr204Cys (Homozygous)	p.Arg367Trp (Homozygous)	p.Pro167Thr (Homozygous)	p.Phe269Ser (Homozygous)	p.Pro213Leu and p.Gly525Arg	
Protein domain		N/A; EMAP-II domain	Aminoacylation domain	N/A	EMAP-II domain	Aminoacylation domain	Anticodon binding domain	Aminoacylation; t-RNA binding domain	
Sex		M	F	M	4F; 8M	5F; 2M	F	1M; 1F	
Age		10 months	24 months	12 months	3 years to 15 years	5 months to 5 years	26 years	15 years; 4.5 years	
IUGR		+	+	−	−	4/7	−	+	8/25 (32%)
Preterm birth		−	+	+	−	3/7	−	−	5/25 (20%)
Failure to thrive		+	+	+	11/12	+	+	+	24/25 (96%)
Short stature		+	+	−	+	5/7	−	+	21/25 (84%)
Neurological and cognitive outcome	Hypotonia	+	+	−	9/12	−	+	+	20/25 (80%)
	Corpus callosum defects	−	−	−	6/9 (thinning)	3/7 (absent myelination)	++ (agenesis)	+ (thinning)	12/22 (55%)
	Microcephaly	+	+	−	+	+	−	−	21/25 (84%)
	Developmental delay	+	+	+	+	+	−	1/2	23/25 (92%)
	Nystagmus	+	−	−	−	6/7	+	−	8/25 (32%)
	Ataxia	−	−	−	7/10	−	−	−	7/23 (30%)
	Seizure	+	−	−	−	1/7	+	−	3/25 (12%)
Pulmonary	Lung disease	−	+	+	1/12	3/7	−	1/2	7/25 (28%)
Facial dysmorphism	Wide nasal bridge	+	−	−	−	−	−	+	3/25 (12%)
	Deep-set eyes	+	−	+	5/7	+	−	+	16/20 (80%)
	Prominent cheeks	+	−	+	−	−	−	+	4/25 (16%)
	Facial weakness	−	−	−	6/10	−	−	1/2	7/23 (30%)
	Microretrognathia with high palate	+	−	−	−	−	−	1/2	2/25 (8%)
	Narrow forehead	+	−	−	−	−	−	−	1/25 (4%)
	Long forehead	−	−	−	−	2/7	−	−	2/25 (8%)
	Flat philtrum	−	−	−	6/7	−	−	−	6/20 (30%)
	Open mouth appearance	−	−	−	6/7	−	−	−	6/20 (30%)
	Hanging columella	−	−	−	5/7	−	−	−	5/20 (25%)
Gastrointestinal	Chronic diarrhea	−	+	−	−	−	−	−	1/25 (4%)
	Vomiting	+	+	−	2/12	4/7	−	+	10/25 (40%)
	Pancreatic insufficiency	−	−	+	−	+	−	−	8/25 (32%)
	Splenomegaly	−	+	−	−	−	−	1/2	2/25 (8%)
Liver	Cholestasis	−	+	+	−	+	+	−	10/25 (40%)
	Hepatomegaly	+	+	+	+	−	−	1/2	16/25 (64%)
	Liver fibrosis	−	+	+	−	3/7	+	−	6/25 (24%)
	Liver steatosis	−	+	+	1/11	5/7	+	+	11/24 (46%)
	Hyperechogenic liver	+	−	−	5/7	−	_	−	6/25 (24%)
Hearing and Vision	Retinitis pigmentosum	−	−	−	−	−	+	−	1/25 (4%)
	Hearing impairment	+	−	−	2/11	+	+	−	11/24 (46%)
Hematology	Anemia	+	+	+	+	3/7	+	−	19/25 (76%)
	Lymphocytosis	+	−	−	−	−	−	−	1/25 (4%)
Renal	Proteinuria	+	−	−	−	4/7	−	−	5/25 (20%)
Infectious diseases	Recurrent infections	−	+	+	1/12	3/7	−	−	6/25 (24%)
Metabolism	Hypoglycemia	−	+	+	−	5/7	+	1/2	9/25 (36%)
	Elevated triglycerides	−	+	−	−	−	+	+	4/25 (16%)
Other features	Primary amenorrhea	N/A	N/A	N/A	−	N/A	+	N/A	1/2 (50%)
	Hypothyroidism	−	−	+	4/9	−	−	−	5/22 (23%)
	Hemangioma	−	+	−	−	−	−	1/2	2/25 (8%)

Our case stands out due to the identification of the YARS1 p.Arg367Trp variant in the patient's father. This particular variant has been previously documented by Averdunk et al. (9) as recurrently homozygous in their study of 12 cases, with 8% (1/12) exhibiting mild hearing impairment (9) Interestingly, our patient failed both the initial and subsequent newborn hearing screens in the left ear. Meanwhile, the patient's father, now 48 years old, encountered adult-onset hearing loss, necessitating the use of hearing aids. Additionally, the paternal grandfather also contends with hearing loss. Notably, hearing impairment is a common feature in other tRNA synthetase mutation disorders, such as LARS2, which leads to Perrault syndrome (18), and NARS2, associated with nonsyndromic sensorineural hearing loss (19). This suggests a potential link between the YARS1 p.Arg367Trp pathogenic variant and hearing impairment, though the precise underlying mechanism remains unclear. Moreover, the patient's father reported concurrent complaints of pain and weakness in his feet. When coupled with the presence of hearing loss, this combination strongly suggests Charcot-Marie-Tooth (CMT). It's worth noting that a heterozygous pathogenic variant in YARS1 has been extensively documented in association with CMT (8, 11, 12). It's important to highlight that none of the previously published cases reported parents with similar symptoms or a diagnosis of CMT. This observation strongly suggests that this pathogenetic variant may indeed be a causative factor for CMT. Furthermore, it raises the possibility of late-onset symptoms associated with this variant or, conversely, incomplete penetrance, where carriers of this variant may not exhibit symptoms.

In contrast, the YARS1 p.Leu261Arg variant, hitherto unreported, is considered a variant of uncertain significance. Interestingly, in close proximity to this position, a homozygous variant, p.Phe263Leu, has been associated with microcephaly, developmental delay, and primordial dwarfism in a female patient (20) suggesting the potential pathogenicity of variants in this region. A summary of clinical phenotypes across different cases is provided in Table 1. This table underscores the variability in phenotypes depending on the specific mutation. However, despite this diversity, no clear genotype-phenotype correlations have been identified. Our case, by adding to the growing number of reported cases, reinforces the notion that IMNEPD2 is a heterogeneous disorder with a spectrum of clinical presentations.

### Molecular mechanism of *YARS1*-associated IMNEPD2

The precise molecular mechanisms driving the association of YARS1 pathogenic variants with IMNEPD2 remain incompletely elucidated. Functional investigations into other ARS variants causing biallelic diseases have generated hypotheses suggesting that diminished levels of functional ARS proteins result in inadequate charging of tRNAs, thereby hampering protein synthesis. This inadequacy becomes particularly pronounced during periods of heightened demand, such as rapid growth and infections. As a response, interventions involving supplemental amino acids have shown promise (21). Estève et al. discovered an 80% reduction in YARS1 protein abundance in their patient's cell line. This finding lends support to the notion that a deficiency in YARS1 protein abundance and activity is implicated in the manifestation of YARS1-associated disorder, thus characterizing it as, at least in part, a loss-of-function disorder (8). It's worth noting that impaired protein translation may not be the sole mechanism contributing to IMNEPD2. The C-terminal EMAPII-like domain of the YARS1 gene, which is dispensable for the aminoacylation activity of YARS1, serves an alternative role as a cytokine with robust chemotactic activity for leukocytes and monocytes. It also triggers the production of tumor necrosis factor-α, myeloperoxidase, and tissue factor (16). Additionally, this domain participates in the assembly of a multisynthetase complex, influencing processes such as angiogenesis, wound healing, glucose metabolism, and neuronal development once secreted from apoptotic cells (9). Consequently, mutations in the EMAPII-like domain can lead to deficiencies in these supplementary functions. Considering the diverse molecular mechanisms associated with different YARS1 variants in causing IMNEPD2, further research is imperative to comprehensively unravel the molecular underpinnings of this disorder and facilitate the development of effective therapeutic strategies.

## Conclusion

The autosomal recessive form of YARS1-associated IMNEPD2 presents as a multisystem disorder with a diverse array of manifestations. Our new case has significantly broadened the phenotypic spectrum associated with this disorder. Notably, in contrast to the majority of case reports, our patient did not exhibit pathogenic variants in the catalytic domain of YARS1. The identification of the novel variant Leu261Arg in our case contributes to the growing pool of reference variants for future studies. Of particular interest is the presentation of symptoms in the patient's father, indicative of Charcot-Marie-Tooth (CMT), suggesting that the pathogenic variant p.Arg367Trp may have the potential to manifest as both autosomal dominant CMT and autosomal recessive IMNEPD2 within the same family. This observation underscores the potential risk of CMT in carriers within the families of IMNEPD2 patients, highlighting the importance of preconception genetic counseling—an aspect not previously recognized.

The concurrent presence of 47, XXY (Klinefelter syndrome) in our patient further underscores the significance of comprehensive genetic testing in cases with intricate clinical phenotypes. Given the scarcity of reported cases and the absence of reports involving children or families with this specific biallelic phenotype, coupled with the rarity of children with a dual diagnosis of Klinefelter syndrome, prognostic predictions for this patient remain challenging. Therefore, extensive long-term follow-up studies are warranted to understand the patient's outcomes. Furthermore, ongoing research to unveil the underlying mechanisms and explore potential therapies, including amino acid supplementation tailored to YARS1-associated autosomal recessive disorders, represents the logical next step in advancing our understanding and management of this condition.

## Data Availability

The original contributions presented in the study are included in the article/Supplementary Material, further inquiries can be directed to the corresponding author.
